# Combined PET/CT with thoracic contrast-enhanced CT in assessment of primary cardiac tumors in adult patients

**DOI:** 10.1186/s13550-020-00661-x

**Published:** 2020-07-06

**Authors:** En-Tao Liu, Tao-Tao Sun, Hao-Jian Dong, Si-Yun Wang, Ze-Rui Chen, Chao Liu, Dan Shao, Zhou-Yang Lian, Qiu Xie, Shu-Xia Wang

**Affiliations:** 1 WeiLun PET Center, Department of Nuclear Medicine, Guangdong Provincial People’s Hospital, Guangdong Academy of Medical Sciences, Room 517, 5/F, Weilun Building of Guangdong Provincial People’s Hospital, 106 Zhongshan Er Road, Guangzhou, 510080 Guangdong People’s Republic of China; 2 Department of Cardiology, Guangdong Provincial People’s Hospital, Guangdong Academy of Medical Sciences, 106 Zhongshan Er Road, Guangzhou, 510080 Guangdong People’s Republic of China; 3 Department of Cardiovascular Surgery, Guangdong Provincial People’s Hospital, Guangdong Academy of Medical Sciences, 106 Zhongshan Er Road, Guangzhou, 510080 Guangdong People’s Republic of China; 4 Department of Pathology and Laboratory Medicine, Guangdong Provincial People’s Hospital, Guangdong Academy of Medical Sciences, 106 Zhongshan Er Road, Guangzhou, 510080 Guangdong People’s Republic of China; 5 Department of Radiology, Guangdong Provincial People’s Hospital, Guangdong Academy of Medical Sciences, 106 Zhongshan Er Road, Guangzhou, 510080 Guangdong People’s Republic of China; 6 Division of Adult Echocardiography, Guangdong Provincial People’s Hospital, Guangdong Academy of Medical Sciences, 106 Zhongshan Er Road, Guangzhou, 510080 Guangdong People’s Republic of China

**Keywords:** PET/CT, ^18^F-Fluorodeoxyglucose (^18^F-FDG), Tomography, X-ray computed, Primary cardiac tumors

## Abstract

**Background:**

^18^F-FDG PET/CT is a key molecular imaging modality to noninvasively assess and differentiate benign and malignant cardiac tumors. However, few benign cardiac tumors can be characterized by increased ^18^F-FDG uptake, which makes differential diagnosis difficult. This study sought to retrospectively evaluate whether combined ^18^F-FDG PET/CT with thoracic contrast-enhanced CT (CECT) helps in assessing primary cardiac tumors in adult patients, compared with CECT or PET/CT alone.

**Methods:**

Forty-six consecutive patients who were diagnosed as primary cardiac tumors were enrolled. All patients underwent ^18^F-FDG PET/CT followed by thoracic CECT before biopsy or surgery. Visual qualitative interpretation and quantitative analysis were performed, and diagnostic performance was evaluated.

**Results:**

More than half (16/29) of benign tumors exhibited with mild ^18^F-FDG uptake. There were significant differences in ^18^F-FDG uptake and the degree of absolute enhancement between benign and malignant tumors (*P* < 0.001). The combination of two modalities improved the specificity from 79 to 93%, the positive predictive value from 73 to 89%, and the accuracy of diagnosis from 85 to 93%. There were significant differences between PET/CT alone or thoracic CECT alone and combined modalities (*P* = 0.034 and *P* = 0.026, respectively). The combination with the optimal SUVmax cutoff value generated 94% sensitivity, 100% specificity, 97% negative predictive values, 100% positive predictive values, and 98% accuracy rates.

**Conclusions:**

Combining ^18^F-FDG PET/C with thoracic CECT significantly improved specificity and accuracy compared to CECT or PET/CT alone in detecting tumors. This combination of diagnostic imaging is effective in differentiating malignant from benign masses.

## Introduction

Primary cardiac tumors are extraordinarily rare—the incidence in autopsies from a meta-analysis is 0.02% [1, 2]. Appropriately 25% of primary cardiac tumors are malignancies, where 95% of these are sarcomas; the rest are lymphomas [3]. Because primary cardiac tumors have nonspecific clinical presentations and imaging features, diagnosis and differential diagnosis is still a challenging issue for physicians and radiologists in clinical practice. Thoracic CECT is commonly used, which can provide morphological information as well as the relationship of tumors with their surrounding tissues. Although the multi-phase scanning technique can describe the enhancement degree and pattern of the tumor, it can not completely distinguish the benign and malignant tumors. ^18^F-Fluorodeoxyglucose (^18^F-FDG) PET/CT has been shown to provide incremental diagnostic information on the basis of conventional imaging (CT and MRI) in the determination of malignancy and staging of cardiac tumors [4]. However, few benign primary cardiac tumors can express slight to moderate ^18^F-FDG uptake, which increases the uncertainty of diagnosis [5–9]. Therefore, we questioned whether the combination of the two modalities (CECT and ^18^F-FDG PET/CT) can improve diagnostic accuracy. The aim of this study was to evaluate the diagnostic accuracy of combined ^18^F-FDG PET/CT with thoracic CECT in the differential diagnosis of primary cardiac tumors.

## Methods

### Study design and population

The local Institutional Review Board approved this retrospective study and waived the requirement for informed consent. Forty-six patients who were diagnosed as primary cardiac tumors were enrolled. The tumor was confirmed by pathological diagnosis or by typical CT signs with following up for 5 years. All patients underwent preoperative ^18^F-FDG PET/CT and thoracic CECT.

### Patient preparation

All patients received dietary preparations with high-fat, low-carbohydrate, protein permitted dietary preparation (two meals, lunch and dinner) and fasted for more than 12 h before PET scanning to suppress physiological myocardial ^18^F-FDG uptake [10–13]. The detailed information on the consumed diet was summarized in Supplementary Materials 1, and the carbohydrate content was less than 2.5 g per meal, which is lower than the 5 g recommended by the Japanese Society of Nuclear Cardiology [14–16]. According to the myocardial metabolism and referring to Williams and Kolodny’s criteria, the myocardial glucose suppression (i.e., the degree of myocardial glucose uptake) was divided into four grades based on visual evaluation [10–12]: grade 0 (negligible uptake), grade 1 (mostly minimal or mild uptake), grade 2 (mostly intense or moderate uptake), and grade 3 (homogeneously intense uptake).

### PET/CT protocol

Image acquisition was performed using a whole-body PET/CT scanner (Biograph HI-REZ 16, Siemens Medical Solution). The equipment detail information was summarized in Supplementary Materials 2. Blood glucose levels (mmol/l) were measured prior to the ^18^F-FDG injection. Blood glucose levels should not be higher than 7 mmol/l; therefore, any patient with a blood glucose level above 7 mmol/l was rescheduled [17–19]. All patients were implanted with 20 G indwelling intravenous catheters (Jierui Medical Product), followed by ^18^F-FDG manual administration with 5.55 MBq/kg. Patients were then instructed to lie on the bed as calmly as possible. Imaging was started 60 min after ^18^F-FDG injection. Detailed scanning and data reconstruction parameters were listed in Supplementary Materials 3.

### Thoracic contrast-enhanced CT protocol

In addition to the PET/CT acquisition, we performed a dual-phase (arterial and venous phase) thoracic contrast-enhanced CT (CECT) (no ECG-gating) with a non-ionic iodinated contrast agent (Iopamiro, 370 mg of iodine/ml, Bracco Sine Pharmaceutical Corp. Ltd., Shanghai, China) on the same PET/CT scanner. The CT component parameters of the PET/CT scanner were summarized in Supplementary Materials 2. The thoracic CECT was achieved with contrast material injected at 4 ml/s (1.0 ml per kg of body weight), followed by an injection of 20 ml of saline at 4 ml/s through a contrast media injector (Ulrich REF XD 2060 or REF XD 2060-Touch, Ulrich Medical, Ulm, Germany). Bolus tracking with a region of interest (ROI) in the descending aorta at the start of the arterial phase thoracic CECT was used to time the CT data acquisition once it reached the threshold value of 100 Hounsfield units (HU). The venous phase thoracic CECT was performed with 50 s delays after contrast agent injection [20]. Detailed scanning and data reconstruction parameters were listed in Supplementary Materials 3.

### Imaging analysis

All PET/CT and thoracic CECT images were transferred to the workstation (Syngo MI workplace, version VA30A, Siemens Healthcare) and reviewed in standard planes. PET/CT images were assessed by two nuclear physicians who received standard training and expertise in PET/CT diagnosis (Dr. TTS and Dr. SYW). They also evaluated the degree of the myocardial ^18^F-FDG uptake and categorized into four grades as previously mentioned. Thoracic CECT images were reviewed by two board-certified radiologists (Dr. DS and Dr. ZYL) with more than 8 years of experience.

### CT diagnostic criteria

For CT image analysis, the diagnostic criteria for benign tumors were as follows: (1) intratumoral calcification, (2) clear and smooth boundary of the tumor, and (3) no enhancement. The diagnostic criteria for malignancy were as follows: (1) right-sided cardiac location (right side of the heart, right atrium, or ventricle), (2) involvement of more than one chamber, (3) extension into the mediastinum or great vessels, (4) broad base of attachment, (5) diameter > 5 cm, and (6) moderate to severe heterogeneous enhancement [21, 22]. The CT attenuation values (HU, Hounsfield units) were measured on transverse images using a manually defined circular ROI with a diameter of 2–10 mm. The ROI was placed in the tumor center at the same location in the pre- and post-contrast (venous phase) enhanced CT images, and intratumoral calcification was avoided. The CT attenuation values represent the average recorded by the previously mentioned radiologists (Dr. DS and Dr. ZYL). The degree of absolute enhancement (△HU) was calculated by subtraction of CT attenuation values obtained with pre- and post-contrast (venous phase) enhanced CT. The following calculation formula was:

$$ \Delta \mathrm{HU}=\mathrm{HUpostcontrast}-\mathrm{HUprecontrast} $$

### PET diagnostic criteria

Maximum and mean standardized uptake values (SUVmax, SUVmean) were measured. The SUVmax and SUVmean represent the average recorded by the previously mentioned nuclear physicians (Dr. TTS and Dr. SYW). According to the optimal cutoff value suggested by Rahbar et al., the diagnostic criteria for malignancy are that the SUVmax of the lesion was greater than 3.5 [4]. We also used the SUVmax corrected for the blood glucose level (SUVgluc) to reduce the effect of blood glucose level on the FDG uptake in tumors. The SUVgluc was calculated by multiplying SUVmax by the blood glucose level (SI unit of blood glucose needs to be multiplied by 18, converted to mg/dl) and dividing it by 100 [23, 24]. The calculation formula used is as follows:

$$ \mathrm{SUVgluc}=\frac{\mathrm{SUVmax}\times \mathrm{blood}\ \mathrm{glucose}\times 18}{100\ } $$

According to the tumor boundaries and the most intense area of tracer accumulation, ROIs were drawn by covering the entire tumors on PET images with proper windows and magnification factors on the axial views. For hypo- or iso-metabolic lesions on ^18^F-FDG PET, ROIs were drawn around the tumor in the axial view based on the anatomic boundaries on CT. Furthermore, we also surveyed the blood pool uptake as background uptake, which was extracted from an ROI placed over the ascending aorta at the level of the carina of the trachea instead of the ventricle. This was done to minimize blurring effects due to cardiac motion and spillover from the myocardium and to reduce the impact of myocardium uptake [25–27]. According to the optimal cutoff value suggested by Rahbar et al., the diagnostic criteria for malignancy are that the SUVmax of the lesion was greater than 3.5 [4]. Tumor-to-background ratios (TBRs) were calculated by dividing the tumors’ SUVmax by the blood pool’s SUVmax. The calculation formula used is as follows:

$$ \mathrm{TBR}=\frac{\mathrm{SUVmax}\ \mathrm{of}\ \mathrm{tumor}}{\mathrm{SUVmax}\ \mathrm{of}\ \mathrm{ascending}\ \mathrm{aorta}\ } $$

### Statistical analysis

The inter-rater reliability of ^18^F-FDG PET/CT and CECT readings was evaluated using Cohen’s kappa (*κ*) and McNemar’s test. The inter-rater reliability of myocardial ^18^F-FDG uptake suppression was evaluated by using weighted kappa (*κ*) and Kendall's Tau-b. We used the linear-by-linear association method for comparing the grade of myocardial ^18^F-FDG uptake suppression between the benign and malignant groups. The Mann-Whitney *U* test or Student’s *t* test was used to compare two independent groups. The receiver operating characteristic (ROC) curve and the area under the curve (AUC) were calculated to assess the ability of ^18^F-FDG PET/CT, thoracic CECT, and the two methods combined. The SPSS Statistics Software (v.23.0, Chicago, IL, USA) and the MedCalc Statistical software (v.15.8, Ostend, Belgium) were used for analysis. A two-tailed probability value of less than 0.05 was considered statistically significant.

## Results

### Population

Forty-six patients presented with a single tumor, a total of 46 tumors, including 29 benign tumors, 15 malignant tumors, and 2 intermediate malignant tumors. The characteristics of the patients are summarized in Table 1.

**Table 1:** Characteristics of patients (*n* = 46)

	All	Benign tumors	Malignant tumors	***P*** value
Number of patients	46	29	17	
Age (years)	44.8 (15.0–71.0)	45.4 (15.0–71.0)	43.7 (20.0–63.0)	0.711
Gender, *n* (%)				0.885
Male	25	16	9	
Female	21	13	8	
Blood glucose level (mmol/l)	5.4	5.2	5.6	0.062
^18^F-FDG injected dose (MBq)	409.8	407.1	414.4	0.792
Size (cm)				
Length	4.2 (0.8–16.9)	3.4 (0.8–8.4)	5.2 (1.8–16.9)	0.006
Width	3.5 (1.1–10.0)	3.4 (1.1–7.9)	4.2 (1.9–10.0)	0.085
Height	3.9 (0.7–14.4)	3.5 (0.7–8.1)	6.9 (3.3–14.4)	0.003
Location				0.923
Atrium	30	18	12	
Left	10	7	3	
Right	20	11	9	
Ventricle	16	11	5	
Left	4	3	1	
Right	12	8	4	
Calcification	6	5	1	
ΔHU	20.5 (1.0–76.0)	17.0 (1.0–65.0)	26.0 (18.0–76.0)	< 0.001
SUVmax_background	2.1 (1.3–3.0)	2.1 (1.3–2.9)	2.2 (1.8–3.0)	0.471
SUVmax_tumor	3.5 (0.8–20.4)	2.7 (0.8–4.9)	6.7 (3.5–20.4)	< 0.001
SUVmean_tumor	2.4 (0.6–8.6)	2.0 (0.6–4.6)	4.3 (2.2–8.6)	< 0.001
SUVmax_Glu_corrected	3.4 (0.7–22.8)	2.5 (0.7–5.7)	6.6 (3.4–22.8)	< 0.001
TBR	1.7 (0.5–11.3)	1.3 (0.5–2.3)	3.2 (1.3–11.3)	< 0.001
Pleural effusion	13	3	10	< 0.001
Pericardial effusion	16	3	13	< 0.001
Treatment and histopathology				
Complete resection	35	28	7	
Partial resection	4		4	
Biopsy only	5		5	
No histopathology	2	1^*^	1^†^	
Newly diagnosed	42	26	16	
Recurrences	4	3	1	
Tumor classification, *n* (%)				
Benign tumors		29		
Myxoma		19		
Hemangioma		4		
Lipoma		3		
Other		3		
Malignant tumors			17	
Sarcoma			12	
Angiosarcoma			6	
Leiomyosarcoma			2	
Other			4	
Lymphoma			2	
Intermediate Malignant tumors^‡^			2	
Other			1	
Metastasis				
Thoracic metastasis			5	
Distant metastasis			1^§^	

^*^One case of lipoma diagnosed by CT, without histopathology

^†^Recurrence of right ventricular myxoid malignant fibrous histiocytoma 1 year after resection, had no operation and no pathology

^‡^Including two cases of intermediate tumors (including 1 Kaposiform hemangioendothelioma and 1 fibrous tumor)

^§^Simultaneous intrathoracic and distant metastases in the one patient

**Fig. 1:**
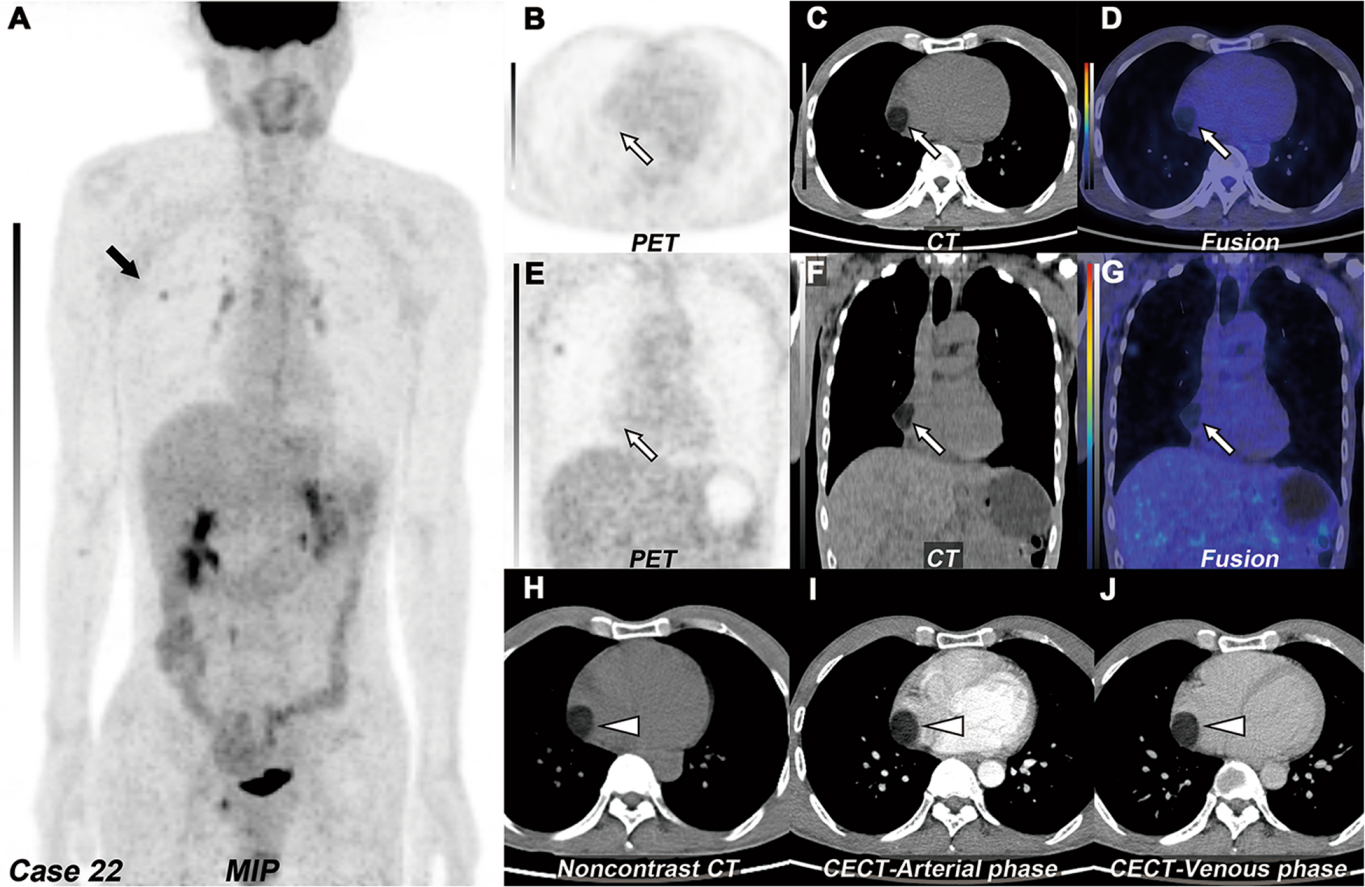
Right atrial lipoma. **a** MIP of ^18^F-FDG PET/CT revealed lung cancer with increased ^18^F-FDG uptake in the right upper lobe (black arrow). **b**–**d** The transverse and **e**–**g** coronal view of selected PET, CT, and fused PET/CT images revealed a well-circumscribed solitary mass, which located in the right atrium (white arrow, SUVmax 0.8). **h** On the non-contrast CT, the tumor presented as sharply margin, ovoid shape with fat density; the CT attenuation value was − 50 HU (white arrowhead). **i**–**j** The transverse view of arterial and venous phases of CECT images revealed no enhancement of the tumor

Of the 46 tumors, one tumor was diagnosed by lipoma due to typical fat attenuation and no enhancement on CECT without any changes during a 6-year follow-up (Fig. 1), and 45 tumors were diagnosed histopathologically. Of these 45 tumors, 41 tumors were newly diagnosed, and 4 tumors were recurrences, of which 3 tumors with myxomas were diagnosed histopathologically again after complete resection (complete re-resection and pathologically confirmed recurrence of myxoma), and the remaining 1 tumor (recurrence of right ventricular myxoid malignant fibrous histiocytoma 1 year after resection) had no operation and no pathology. Among these tumors (41/45) newly diagnosed by histopathology, all benign tumors (25/25) underwent complete tumor resection, whereas seven of 16 malignant tumors underwent complete tumor resection, four malignant tumors underwent partial tumor resection, and five malignant tumors underwent biopsy only.

### Agreement on visual assessment

By visual evaluation of benign and malignant lesions, Cohen’s kappa (*κ*) value and of ^18^F-FDG PET/CT (Dr. TTS and Dr. SYW) and CECT (Dr. DS and Dr. ZYL) were 0.870 and 0.867, respectively (*P* < 0.001). The McNemar’s test found a *P* value > 0.05 (*P* = 0.25 and *P* = 1.00, respectively). By visual evaluation, the suppression of myocardial ^18^F-FDG uptake, weighted kappa (*κ*) values of the grade of myocardial ^18^F-FDG uptake suppression were 0.785 (*P* < 0.001, 95% CI 0.659–0.911), and Kendall’s Tau-b coefficient was 0.838 (*P* < 0.001).

### The suppression of myocardial ^18^F-FDG uptake on quantitative assessment

The grade of myocardial ^18^F-FDG uptake suppression did not differ statistically significantly between the benign and malignant groups (linear-by-linear association value, 2.840, *P* = 0.092; 3.512, *P* = 0.061, respectively).

### Baseline characteristics

The baseline characteristics of the study population were summarized in Table 1. There were no statistically significant differences in age, gender, blood glucose level, ^18^F-FDG injected dose, or tumor location between benign and malignant groups.

### ^18^F-FDG PET/CT and thoracic CECT

**Fig. 2:**
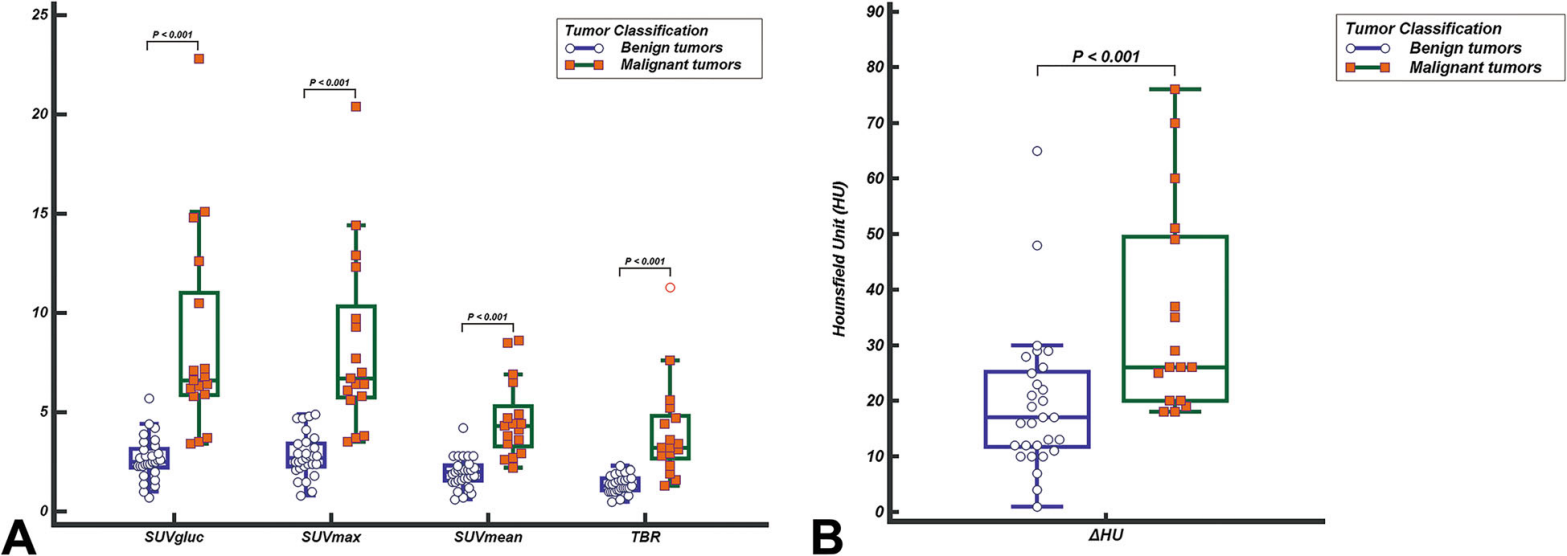
Median comparison of ^18^F-FDG uptake (**a**) and the enhancement (**b**) of tumors. There was a statistically significant difference in the median between benign and malignant groups (*P* < 0.001)

**Fig. 3:**
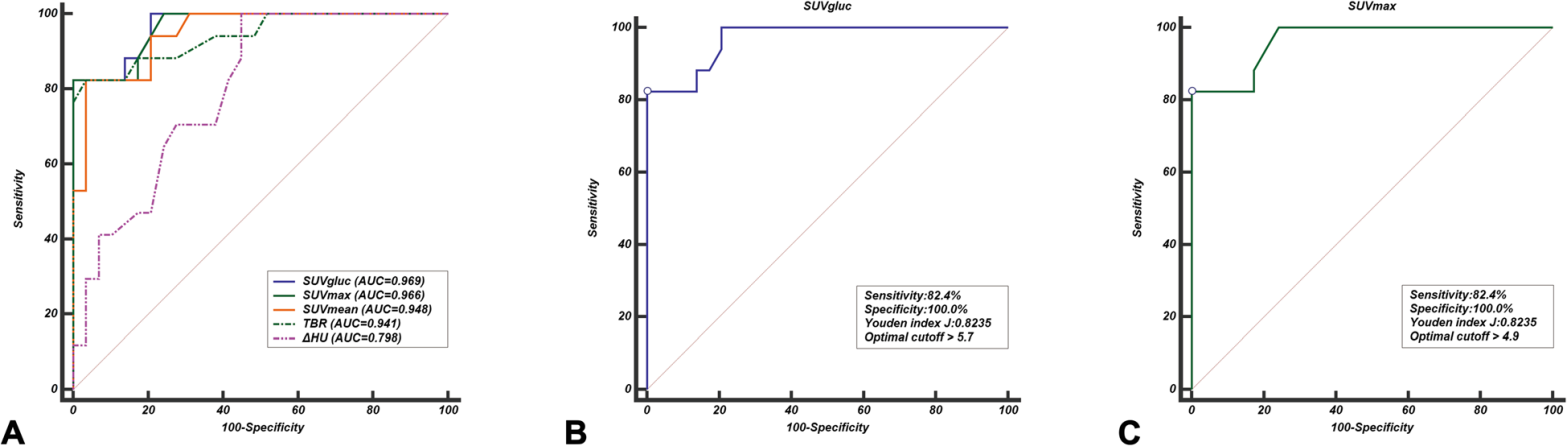
ROC curves of the parameter of ^18^F-FDG uptake and the enhancement. **a** The SUVgluc had a higher AUC value (0.969) discriminating between malignant and benign tumors than other parameters. **b** The SUVgluc cutoff value of 5.7 and **c** the SUVmax cutoff value of 4.9 yield the same 82.4% sensitivity and 100.0% specificity

The median values of benign versus malignant tumors were as follows: SUVmax, 2.7 versus 6.7; SUVmean, 2.0 versus 4.3; SUVgluc, 2.5 versus 6.6; TBR, 1.3 versus 3.2; and ΔHU, 17.0 versus 26.0. There were statistically significant differences in the above medians between benign and malignant groups (Fig. 2). By ROC analysis, the best parameter corresponded to SUVgluc which had the highest AUC (*P* < 0.001, 95% CI 0.870–0.998), and the optimal cutoff values of SUVgluc and SUVmax were 5.7 and 4.9, respectively, which could generate 82.4% sensitivity and 100% specificity (Fig. 3). Thoracic CECT alone showed 82% sensitivity, 83% specificity, and 83% diagnostic accuracy. PET/CT alone showed 94% sensitivity, 79% specificity, and 85% diagnostic accuracy. And the combination of two modalities showed 94% sensitivity, 93% specificity, and 93% diagnostic accuracy (Table 2). There were significant differences between PET/CT alone or thoracic CECT alone and combined two modalities (*P* = 0.034 and *P* = 0.026, respectively). The combination with the optimal SUVmax cutoff value of 4.9 generated the optimal sensitivity, specificity, negative predictive values, positive predictive values, and accuracy rates (Table 2).

**Table 2:** Diagnostic performance of various parameters

Feature	Sensitivity (%)	Specificity (%)	Positive predictive value (%)	Negative predictive value (%)	Accuracy (%)
PET/CT alone*	94	79	73	96	85
Thoracic CECT alone	82	83	74	89	83
Combined two modalities	94	93	89	96	93
SUVgluc cutoff					
> 5.7	82	100	100	91	93
SUVmax cutoff					
> 4.9	82	100	100	91	93
> 5.2 [28]	82	100	100	91	93
> 3.5 [4]	94	79	73	96	85
Combined two modalities with cutoff > 4.9	94	100	100	97	98

*The diagnostic cutoff is according to Rahbar et al.[4]

### Other findings

**Fig. 4:**
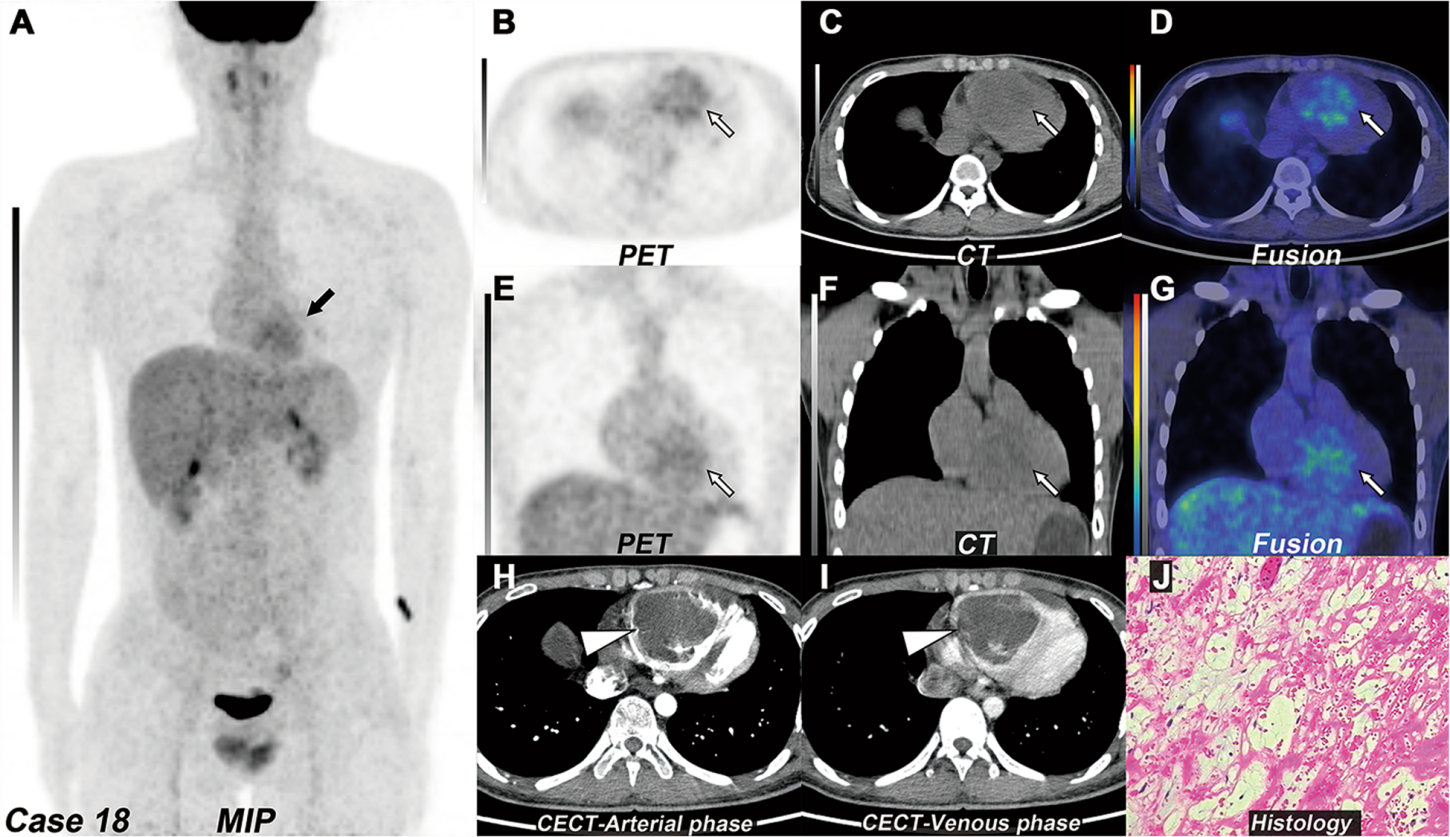
Right ventricular myxoma. **a** MIP of ^18^F-FDG PET/CT revealed a lesion with increased ^18^F-FDG uptake in the heart region (black arrow). **b**–**d** The transverse and **e**–**g** coronal view of selected PET, CT, and fused PET/CT images revealed a big mass with increased ^18^F-FDG uptake (white arrow, SUVmax 4.1), which located in the right ventricle. **c** and **f** On the non-contrast CT, the tumor presented homogeneous hypodensity, which was lower than the blood pool. **h** and **i** The transverse view of arterial and venous phases of CECT images revealed a huge filling defect in the right ventricle with mild enhancement in the venous phase (white arrowhead). **j** The histopathology revealed myxoma (H&E, magnification, × 400)

**Fig. 5:**
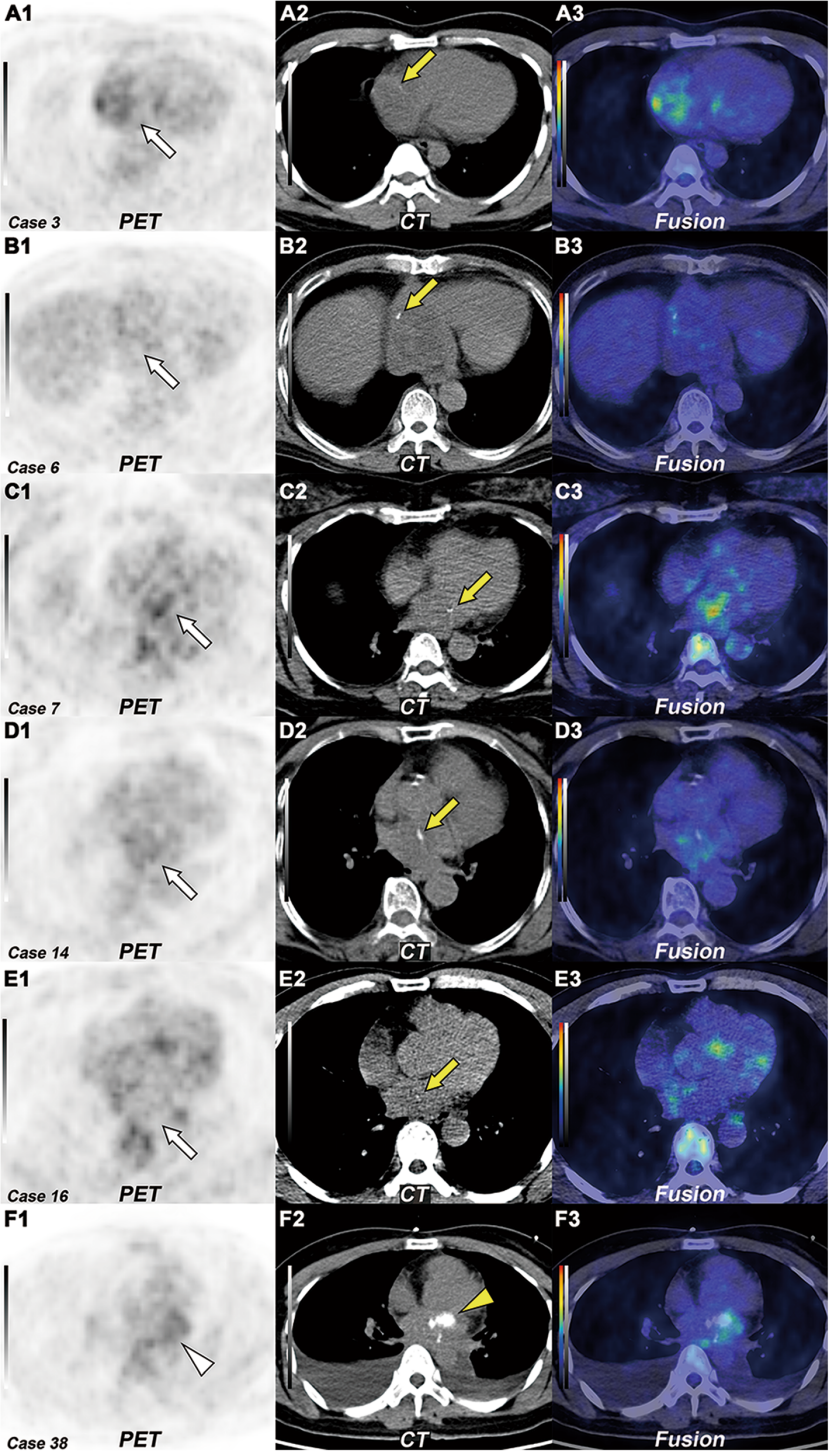
Intratumoral calcification in myxoma and myxosarcoma. **A**–**E** The transverse view of selected PET (1), CT (2), and fused PET/CT (3) images revealed five myxomas with none to mildly increased ^18^F-FDG uptake (white arrow, SUVmax 2.2–4.7). On the non-contrast CT (**A2**–**E2**), the tumor presented punctate calcification (yellow arrow). **F1**–**F3** The transverse view of selected PET, CT, and fused PET/CT images revealed a myxosarcoma with mildly increased ^18^F-FDG uptake (white arrowhead, SUVmax 3.5). On the non-contrast CT (**F2**), the tumor presented nodular calcification (yellow arrowhead)

**Fig. 6:**
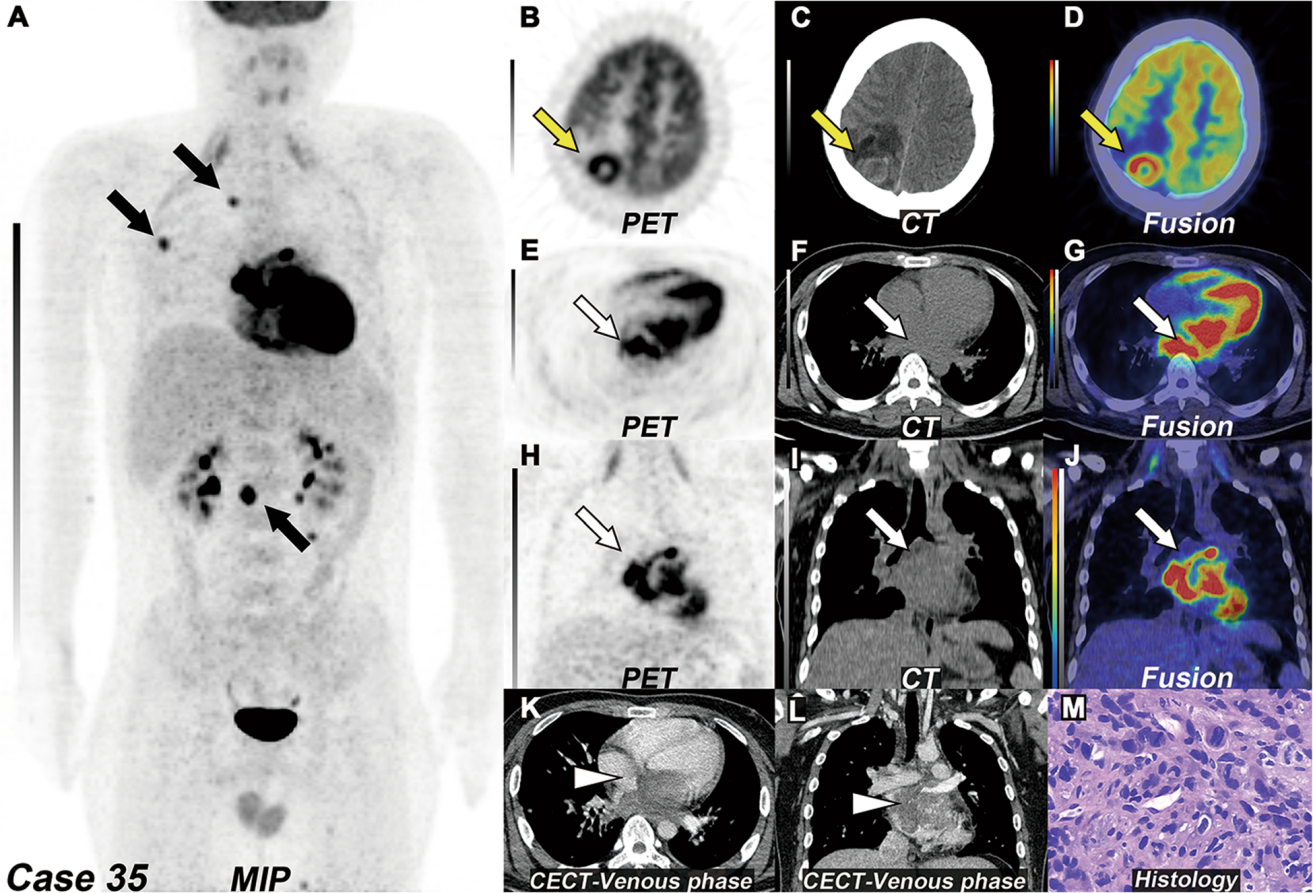
Left atrial angiosarcoma with multiple metastases. **a** MIP of ^18^F-FDG PET/CT revealed multiple bones and muscle metastasis with focal increased ^18^F-FDG uptake (black arrow, SUVmax 5.6–10.0) and severely increased ^18^F-FDG uptake by myocardium, due to lack of dietary preparation. **b**–**d** The transverse view of selected PET, CT, and fused PET/CT images revealed a brain metastasis with increased ^18^F-FDG uptake in the right parietal lobe (yellow arrow). **e**–**g** The transverse and **h**–**j** coronal view of selected PET, CT, and fused PET/CT images revealed a big mass with severely increased ^18^F-FDG uptake (white arrow, SUVmax 14.4), which located in the left atrium. **k** The transverse and **l** coronal view of CECT images revealed an irregular mass with moderately to intensely enhancement in the venous phase (white arrowhead). **m** The histopathology of biopsy of one of the muscle metastases revealed angiosarcoma (H&E, magnification, × 400)

Sixteen of 29 benign tumors showed visually increased ^18^F-FDG uptake, and 22 tumors showed TBR higher than 1. Eleven of 19 myxomas showed visually increased FDG uptake, and 15 cases showed TBR higher than 1 (Fig. 4). Six tumors demonstrated foci calcification on NE-CT, including five myxomas and one myxosarcoma (Fig. 5). There were statistically significant differences in pericardial and pleural effusions between benign and malignant groups. More than half (10~13/17) of malignant tumors were accompanied by pericardial and pleural effusions. Five cases of malignant tumors were combined with intrathoracic metastasis (pericardium, mediastinum, lung), and one case was combined with distant metastasis (Fig. 6).

## Discussion

To our knowledge, the present study includes by far the largest number of adult patients with primary cardiac tumors assessed using ^18^F-FDG PET/CT scan [4, 28–30]. In this study, the ^18^F-FDG uptake level (SUVmax, SUVmean, SUVgluc, and TBR) in the primary malignant cardiac tumor was more than twice (2.2–2.6:1) that of the primary benign cardiac tumor, and the difference was statistically significant (*P* < 0.001). Although ^18^F-FDG PET/CT could provide the metabolic rate of glycolysis in tumors, which is helpful for the diagnosis of malignancy, it lacks effective morphological information for hypo- or iso-metabolic tumors on ^18^F-FDG PET/CT. Moreover, in this current study, sixteen of 29 benign tumors showed visually increased ^18^F-FDG uptake; twenty-two of 29 tumors showed TBR higher than 1, of which eleven of 19 myxomas showed visually increased ^18^F-FDG uptake and fifteen of 19 myxomas showed TBR higher than 1. This presents a challenge in identifying benign and malignant cardiac tumors using ^18^F-FDG PET/CT alone. Therefore, thoracic CECT is helpful in determining tumor localization, delineating the shape of the tumor, and assessing for tumor invasion of adjacent structures. The arterial phase could demonstrate the filling defect of the tumor and delineate the boundary of the tumor, whereas the venous phase could evaluate the enhancement of the tumor and describe the enhancement pattern of the tumor. The combination of thoracic CECT improved the accuracy of diagnosis from 85 to 93%, especially for benign cardiac tumors. There were significant differences between PET/CT alone or thoracic CECT alone and combined two modalities (*P* = 0.034 and *P* = 0.026, respectively).

**Fig. 7:**
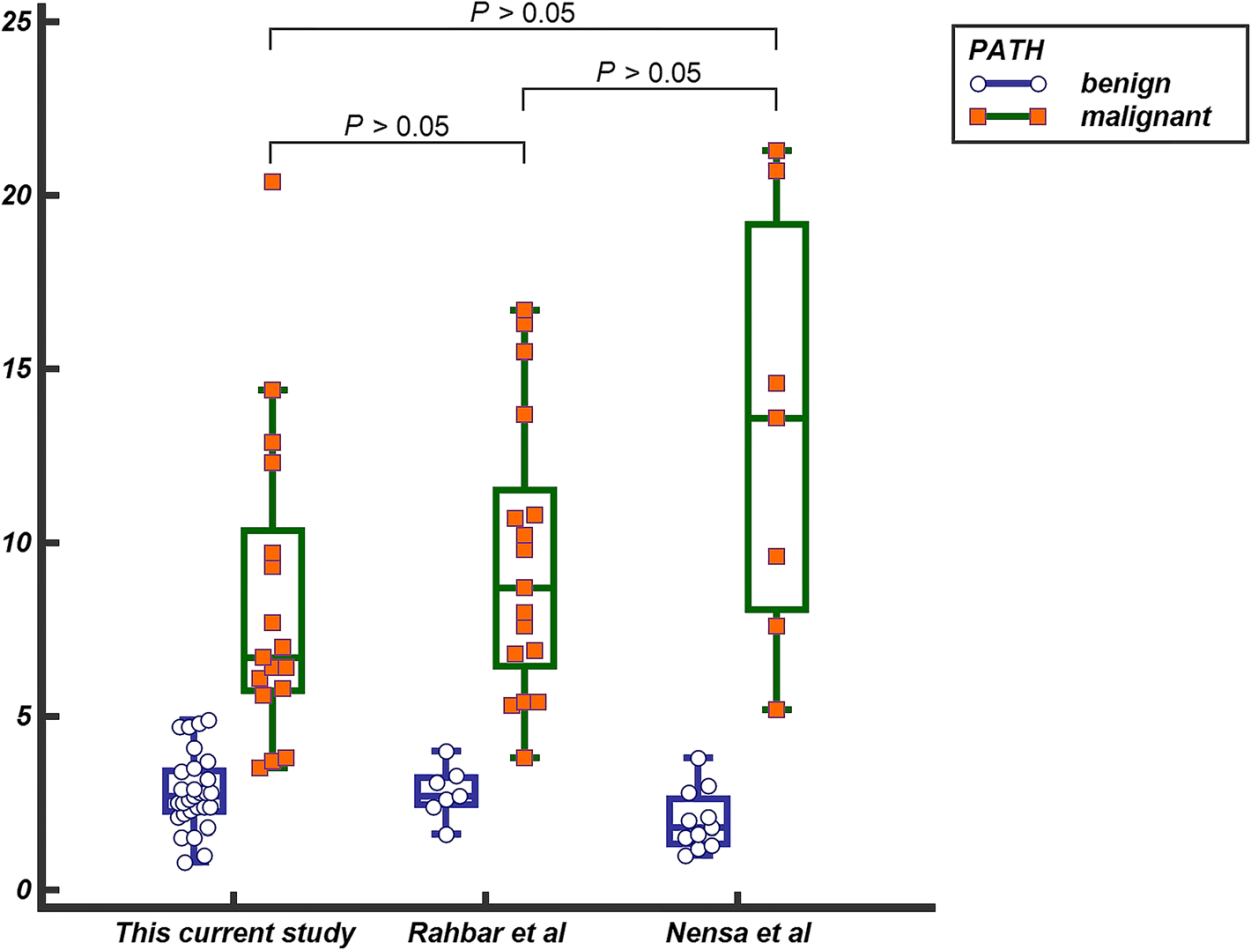
Comparison of this current study with Rahbar et al.’s [4] and Nensa et al.’s [28] study. There was no statistically significant difference in the median (SUVmax) of benign and malignant groups in the above three groups (*P* > 0.05)

In our PET diagnostic criteria, we referred to the cutoff value of 3.5 from Rahbar et al. as a reference index, which is not limited to this index [4]. We also calculated the sensitivity and specificity of our data by using the cutoff value of 5.2 from Nensa et al. [28]. The combination of two modalities (PET/CT and thoracic CECT) with the optimal SUVmax cutoff value of 4.9 generated the optimal sensitivity, specificity, negative predictive values, positive predictive values, and accuracy rates (Table 2), higher than that obtained by applying the above 3.5 or 5.2 cutoff values to our data, similar to the results (cutoff > 4.6) reported by Rahbar et al. but still lower than that reported by Nensa et al. [28]. We also compared the data from the above two studies with our data and found no differences between the same types of tumors (Fig. 7), although the above study included cardiac metastatic tumors. The reason we speculated was that as a supplement of characterizing tumor morphology, MRI is a more helpful tool in improving the sensitivity and specificity of diagnosis due to high-tissue resolution. Zhu et al.’s and Yaddanapudi et al.’s studies also showed that combining PET/CT with MRI or hybrid PET/MRI both have strong potential in the diagnosis of cardiac and paracardiac masses with histopathologic correlation [29, 31]. However, there is still a need to expand the sample size and include multi-center studies [32, 33].

Unlike other studies, intratumoral calcification was used as a diagnostic criterion for benign cardiac tumors in the current study. Previous studies have reported that nearly 75% of primary cardiac tumors are benign, with the majority (more than 50%) in adults being myxomas [1, 34, 35]. Visual intratumoral calcification occurs in 30–50% of myxoma cases on non-contrast CT [32, 33, 36]. Thus, radiographic visualization of intratumoral calcification should consider the possibility of myxoma [32]. In this study, 6 cases of intratumoral calcification were found, including 5 cases of myxoma and 1 case of myxosarcoma. Therefore, we recommend that myxoma should be considered as the first diagnosis if the tumor is accompanied by visual intratumoral calcification, and there is no increased ^18^F-FDG uptake.

The main reasons for misdiagnosis were analyzed as follows: firstly, some benign tumors had a slightly increased uptake, as mentioned previously. Secondly, the diameter of some benign tumors was greater than 5 cm. Nine of 29 benign tumors had a maximum diameter of more than 5 cm. These two factors were the main factors that interfered with the correct diagnosis. It is therefore inappropriate to differentiate benign from malignant cardiac tumors by tumor size.

For malignant cardiac tumors, in this study, twelve of 17 malignant tumors were sarcomas, including 6 angiosarcomas and 5 cases with lung and distant metastasis (brain, bone, and muscle). It was reported that about half of patients with cardiac angiosarcomas were accompanied by systemic metastases; most of them were lung metastasis [37–39]. PET/CT system information can not only provide metabolic information of the primary tumor but also help to describe the tumor invasion range, evaluate the tumor metastasis, detect distant metastasis, and guide biopsy [40]. As the 35th case in this study, the whole-body information showed the lesion of the erector spinae muscle. We carried out a pathologic biopsy of the lesion to confirm the diagnosis of cardiac angiosarcoma based on whole-body information (Supplementary Materials 4).

### Limitations of our study

The main limitation of this present study is its retrospective design, which is associated with selection bias due to a lack of randomization. Another limitation stems from the fact that some critically ill patients in this study were excluded because they were unable to undergo biopsy and had no follow-up. Thus, the study population was skewed to patients who tended to be less ill and more likely to undergo resection or biopsy. This selection bias suggests that these findings may not be extended to the general population, although this too is speculative. Other further limitations included the increase in radiation exposure form the additional thoracic CECT scan as well as the lack of oncological follow-up data and further analysis. Further prospective assessment in a large sample size should be performed to confirm these preliminary results and evaluate the performance of combined PET/CT and thoracic CECT in primary cardiac tumors. Future research with long-term and comprehensive oncological follow-up and comparison of the different pathological types, intracardiac thrombosis, benign cardiac tumors, malignant primary, and secondary tumors are needed to assess the repeatability of combined PET/CT and thoracic CECT and uptake variation of lesions.

## Conclusions

The use of both combined thoracic CECT with ^18^F-FDG PET/CT significantly improved specificity and accuracy compared to CECT or PET/CT alone in detecting tumors. This combination of diagnostic imaging is effective in differentiating malignant from benign masses.

## Supplementary information

**Additional file 1:.** Supplementary Material 1: Dietary Preparation

**Additional file 2:.** Supplementary Material 2: PET scanner

**Additional file 3:.** Supplementary Material 3: Detailed scanning parameters

**Additional file 4:.** Supplementary Material 4: Indication of the biopsy site

## Data Availability

The datasets used and/or analyzed during the current study are available from the corresponding author on reasonable request.
