# Supplementary material for: Combined PET/CT with thoracic contrast-enhanced CT in assessment of primary cardiac tumors in adult patients
Source: EJNMMI Res. 2020 Jul 6;10:75. doi: 10.1186/s13550-020-00661-x (PMC7338301; doi:10.1186/s13550-020-00661-x)
Supplement: Supplementary file 1 — Additional file 1:. Supplementary Material 1: Dietary Preparation [file 13550_2020_661_MOESM1_ESM.docx]

**DIETARY PREPARATION**

1. Dietary preparation methods
   1. The dietary preparation menu
      1. High fat, Low-carbohydrate, and protein permitted diet consisted of 100 g of fried egg cooked in 10-20 ml of cooking oil per meal (All Nutrition Facts from <https://www.fatsecret.com/>).

Energy 811.696 kJ

Protein 13.56 g

Fat 14.69 g

Carbohydrate 0.93 g

100 g of cooking oil contains 100 g of fat.

- - 1. Recipe: Five fried eggs and some pure water for each meal.
  1. Preparation methods: All patients received two special meals (above recipe) and fasted for more than 12 hours the day before PET scanning.
  2. Evaluation criteria of myocardial glucose suppression
     1. According to the myocardial metabolism and referring to Williams and Kolodny’s criteria, the myocardial glucose suppression (i.e., the degree of myocardial glucose uptake) was divided into four grades based on visual evaluation: ^1–3^

Grade 0 (negligible uptake),

Grade 1 (mostly minimal or mild uptake),

Grade 2 (mostly intense or moderate uptake),

Grade 3 (homogeneously intense uptake).

- - 1. Statistic evaluation: The inter-rater reliability of myocardial ^18^F-FDG uptake suppression was evaluated by using weighted kappa (κ) and Kendall's Tau-b. We used the linear-by-linear association method for comparing the grade of myocardial ^18^F-FDG uptake suppression between the benign and malignant groups.

1. Results:
   1. Agreement on visual assessment

By visual evaluation, the suppression of myocardial ^18^F-FDG uptake, weighted kappa (κ) values of the grade of myocardial ^18^F-FDG uptake suppression were 0.785 (*P* < 0.001, 95% CI 0.659-0.911), and Kendall's Tau-b coefficient was 0.838 (*P* < 0.001).

- 1. The suppression of myocardial ^18^F-FDG uptake on quantitative assessment

The grade of myocardial ^18^F-FDG uptake suppression did not differ statistically significantly between the benign and malignant groups (linear-by-linear association value, 2.840, *P* = 0.092; 3.512, *P* = 0.061, respectively).

Notes:

According to the consumption of 6.276 kJ per person per day, and 0.811696 kJ per 100 g of fried eggs, each person needs 773 g of fried eggs a day. Three meals a day, it is equivalent to eating 257.66 g of fried eggs per meal (5 fried eggs).

According to the Japanese Society of Nuclear Cardiology Recommendations, in the low-carbohydrate diet, the carbohydrate content is less than 5 g per meal.^4–6^ In accordance with the preparation method we used, the carbohydrate content is less than 2.5 g per meal.

**REFERENCE**

1. Williams G, Kolodny GM. Suppression of myocardial 18F-FDG uptake by preparing patients with a high-fat, low-carbohydrate diet. AJR.2008;190:W151-156. doi: 10.2214/AJR.07.2409
2. Harisankar CN, Mittal BR, Agrawal KL, Abrar ML, Bhattacharya A. Utility of high fat and low carbohydrate diet in suppressing myocardial FDG uptake. J Nucl Cardiol. 2011;18:926-36. doi: 10.1007/s12350-011-9422-8
3. Demeure F, Hanin FX, Bol A, Vincent MF, Pouleur AC, Gerber B, et al. A randomized trial on the optimization of ^18^F-FDG myocardial uptake suppression: implications for vulnerable coronary plaque imaging. J Nucl Med. 2014;55:1629-35. doi: 10.2967/jnumed.114.138594
4. Ohira H, Tsujino I, Yoshinaga K. ^18^F-Fluoro-2-deoxyglucose positron emission tomography in cardiac sarcoidosis. Eur J Nucl Med Mol Imaging. 2011;38:1773-83. doi: 10.1007/s00259-011-1832-y
5. Ishida Y, Yoshinaga K, Miyagawa M, Moroi M, Kondoh C, Kiso K, et al. Recommendations for (18)F-fluorodeoxyglucose positron emission tomography imaging for cardiac sarcoidosis: Japanese Society of Nuclear Cardiology recommendations. Ann Nucl Med. 2014;28:393-403. doi: 10.1007/s12149-014-0806-0
6. Kumita S, Yoshinaga K, Miyagawa M, Momose M, Kiso K, Kasai T,et al. Recommendations for 18F-fluorodeoxyglucose positron emission tomography imaging for diagnosis of cardiac sarcoidosis-2018 update: Japanese Society of Nuclear Cardiology recommendations. J Nucl Cardiol. 2019;26:1414-1433. doi: 10.1007/s12350-019-01755-3
