# Supplementary material for: Combined PET/CT with thoracic contrast-enhanced CT in assessment of primary cardiac tumors in adult patients
Source: EJNMMI Res. 2020 Jul 6;10:75. doi: 10.1186/s13550-020-00661-x (PMC7338301; doi:10.1186/s13550-020-00661-x)
Supplement: Supplementary file 2 — Additional file 2:. Supplementary Material 2: PET scanner [file 13550_2020_661_MOESM2_ESM.docx]

***PET/CT scanner (Siemens Biograph HI-REZ 16, Siemens Medical Solution) detail information (Table 1. and Table .2)***

| **Table 1. PET component Parameters of the PET/CT scanner** | |
| --- | --- |
| **Characteristic** | **Value** |
| Crystal material | Lutetium oxyorthosilicate (LSO) |
| Crystal Dimensions | 4.0 × 4.0 × 20 mm |
| Crystals Per Detector Block | 169 |
| Total Detector Blocks | 144 |
| Detector Ring Diameter | 830 mm |
| Detectors Per Ring | 624 |
| Number of Detector Rings | 39 |
| Total Number of Detectors | 24336 |
| Transaxial Field of View | 585 mm |
| Axial Field of View | 162 mm |
| Coincidence time resolution | 500 ps |
| Coincidence window | 4.5 ns |
| Reconstruction time | < 2 min/bed |
| System energy resolution | < 15% FWHM |
| Scatter fraction (425 keV LLD) | < 36% |
| Acquisition modes | Static, Multi-bed |
| **Table 2. CT component Parameters of the PET/CT scanner** | |
| **Characteristic** | **Value** |
| Aperture | 700 mm |
| Scan field | 500 mm |
| Rotation time | 0.42, 0.5, 0.75, 1.0, 1.5 s |
| Tube current | 28 - 500 mA |
| Tube voltages | 80, 120, 140 kV |
| Number of detector rows | 24 |
| Elements | 16128 |
| Channels per slice | 1344 |
| Number of projections (/360°) | Up to 2320 |
| Reconstructed slice widths | 0.6, 0.75, 1.0, 1.5, 2.0, 3.0, 4.0, 5.0, 6.0, 7.0, 8.0, 10.0 mm |
| Slice increment | 0.1 - 10.0 mm |
| Pitch factor (volume pithch) | 0.5 - 2.0 (1 - 32) |
| Spiral scan time max | 100 s |
