# Supplementary material for: Combined PET/CT with thoracic contrast-enhanced CT in assessment of primary cardiac tumors in adult patients
Source: EJNMMI Res. 2020 Jul 6;10:75. doi: 10.1186/s13550-020-00661-x (PMC7338301; doi:10.1186/s13550-020-00661-x)
Supplement: Supplementary file 3 — Additional file 3:. Supplementary Material 3: Detailed scanning parameters [file 13550_2020_661_MOESM3_ESM.docx]

**DETAILED SCANNING AND DATA RECONSTRUCTION PARAMETERS**

- ***PET***

A CT scout scan was first acquired, covering the range from the apex of the skull to the upper-thighs. Following this a low-dose CT transmission scan (slice thickness: 5 mm, tube voltage: 120kV, tube current: 50 mAs, pitch: 0.75:1) was performed without intravenous or oral contrast agent, followed by a PET emission scan of 3-min per bed position in 3-dimensional mode, where the PET bed overlap was 30%. During the scan, the patients were asked to lie supine on the scanning bed with the arms at their sides. The static PET data were reconstructed with an iterative ordered subsets expectation maximization (OSEM) algorithm, using the following parameters: 4 iterations, 8 subsets, 168 × 168 matrix, and DFOV 68.3 cm, 5 mm full-width-at-half-maximum (FWHM) Gaussian post-smoothing filter. The CT data were used for attenuation correction and anatomic localization, and the images were reconstructed using a filter-back-projection algorithm.

- CECT

The CECT acquisition parameters were as follows: tube voltage: 120kV, tube current: 140 mAs, pitch: 1.35:1, and slice thickness: 5 mm. The thoracic CECT images were reconstructed by using a standard kernel.
