# Supplementary figures and images for: Combined PET/CT with thoracic contrast-enhanced CT in assessment of primary cardiac tumors in adult patients
Source: EJNMMI Res. 2020 Jul 6;10:75. doi: 10.1186/s13550-020-00661-x (PMC7338301; doi:10.1186/s13550-020-00661-x)

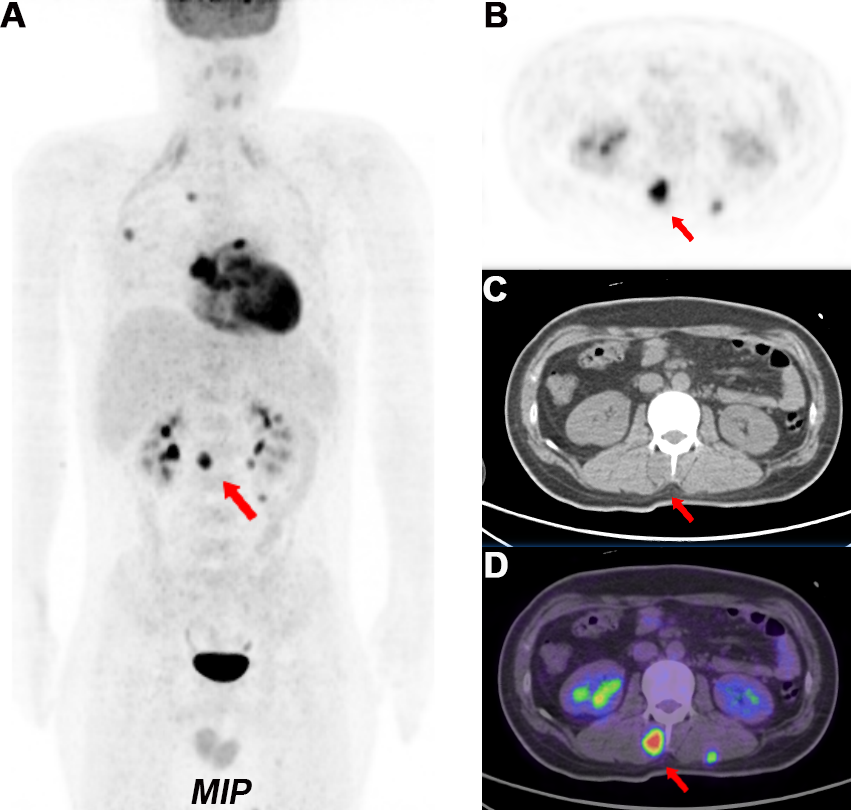

Supplement: Supplementary file 4 — Additional file 4:. Supplementary Material 4: Indication of the biopsy site [file 13550_2020_661_MOESM4_ESM.tif]
